# R263K-associated dolutegravir resistance on tenofovir, lamivudine and dolutegravir: Implications for earlier genotyping and regimen optimisation

**DOI:** 10.4102/sajid.v41i1.790

**Published:** 2026-03-18

**Authors:** Sirashen Naidoo

**Affiliations:** 1 Bheki Mlangeni District Hospital, Soweto, South Africa

**Keywords:** dolutegravir resistance, HIV-1 subtype C, antiretroviral therapy, R263K, functional monotherapy, salvage regimen, South Africa, case report

## Abstract

A South African woman receiving tenofovir, lamivudine and dolutegravir (TLD) with good adherence, developed virological failure with the integrase mutation R263K. Viral suppression was achieved after rapid intensification to dolutegravir 50 mg twice daily combined with atazanavir with ritonavir (ATV/r), with continuation of the nucleoside backbone (dual-anchor regimen). This case highlights the need for earlier resistance testing and specialist-guided salvage therapy for subtype C in resource-limited settings.

**Contribution:**

Archived nucleoside reverse transcriptase inhibitor (NRTI) resistance may render the background drugs functionally inactive, creating functional DTG monotherapy that can select for R263K in HIV-1 subtype C. Swift escalation to salvage therapy may mitigate viraemia. Algorithms should be formulated to warrant earlier genotyping and promote rapid efforts to limit resistance accumulation in patients with documented adherence.

## Background

Dolutegravir (DTG) is highly efficacious and has a strong genetic barrier to resistance, becoming the anchor to first- and second-line regimens in South Africa from 2019. Despite this, DTG resistance has been documented, particularly when paired with inactive nucleoside reverse transcriptase inhibitor (NRTIs).^1,2,3,4^ In suspected or confirmed integrase inhibitor resistance, the recommended approach is dual-anchor therapy that combines a boosted protease inhibitor with an integrase inhibitor, alongside an optimised NRTI backbone.^5^

## Case presentation

The patient (MM) is a 36-year-old woman residing in Soweto who was diagnosed with HIV in 2014. She is unsure of the exact month of antiretroviral therapy (ART) initiation and does not recall the initial regimen; contemporaneous clinic records were unavailable. Earlier records show that she was virally suppressed in 2018; however, social circumstances led to stopping treatment. In 2022, she began tenofovir, lamivudine and dolutegravir (TLD), comprising tenofovir (TDF) 300 mg, lamivudine (3TC) 300 mg and dolutegravir (DTG) 50 mg. Objective adherence measures included consistent pharmacy refills and on-time clinic follow-ups. She also used daily phone alarms as a reminder strategy, confirmed during follow-up. Despite these measures, her cluster of differentiation 4 (CD4) count remained below 20 cells/µL, and her viral load persistently exceeded 100 000 copies/mL.

She was diagnosed with cryptococcal meningitis in 2023, which was complicated by epilepsy. Management was initiated as per national guidelines, and she began sodium valproate, rendering her seizure free. Serum cryptococcal antigen remained positive, without clinical or radiological evidence of active disease.

Resistance testing in November 2024 demonstrated mutations K70Q, M184V and Y115F (NRTI), V106M and V179D (non-nucleoside reverse transcriptase inhibitor) and R263K (integrase). She was escalated to a dual-anchor regimen consisting of dolutegravir 50 mg twice daily plus atazanavir with ritonavir (ATV/r), with continuation of TDF/3TC. At the next routine visit, her viral load improved to below 20 copies/mL with modest recovery of CD4 to 34 cells/µL.

## Outcome and follow-up

Following intensification, the patient maintained near-complete suppression with immune recovery on continued specialist follow-up.

## Discussion

Although DTG has a high genetic barrier to resistance, treatment failure can develop when accompanying NRTIs are compromised despite good adherence. Case reports and literature underscore that DTG is not immune to resistance despite its durability.^1,4^ In this case, the NRTI backbone was likely inactivated by archived NRTI mutations (M184V, K70Q and Y115F), leaving DTG as the only fully active agent. Acquisition of the R263K integrase mutation was a consequence of ongoing selective pressure with failure to suppress viraemia, consistent with functional monotherapy.^1,4^

The patient reports ART initiation in 2014 but cannot recall the exact timing or initial regimen, and contemporaneous clinic records were unavailable. Given the South African first-line policy during that period, an efavirenz-based regimen such as tenofovir, lamivudine and efavirenz (TLE) or tenofovir, emtricitabine and efavirenz (TEE) is plausible, which could explain the archived non-nucleoside reverse transcriptase inhibitor (NNRTI) (V106M, V179D) and NRTI mutations. This remains presumptive because of missing documentation.

R263K attenuates antiviral efficacy by diminishing DTG binding affinity to integrase. R263K impairs integrase function and reduces viral fitness. If viraemia persists under dolutegravir pressure, particularly with inactive companion NRTIs, ongoing replication may select additional integrase mutations such as G118R, increasing resistance and partially offsetting the fitness cost. This explains how reduced fitness can coexist with persistent viraemia and progressive resistance evolution.^4^ Similar patterns have been described in real-world settings. DTG failure is reported in Malawi and Brazil, typically in patients with advanced disease and prolonged viraemia on TLD accompanied by NRTI resistance and, occasionally, integrase mutations such as G118R or E138K.^2,3^ Together with earlier reports, these cases support the concept that the primary risk factor for emergent DTG resistance is persistent viral replication superimposed on inactive companion drugs.^1,4^

Our case highlights management principles pertinent to high-burden settings. Firstly, patients on TLD with documented adherence and persistently high viral loads should prompt earlier resistance testing. In this scenario, the expected resistance pattern is NRTI resistance (often including M184V with additional thymidine analogue or related mutations) with preserved integrase susceptibility; however, functional DTG monotherapy can select for integrase mutations such as R263K or G118R. Secondly, therapeutic inertia by the clinician is detrimental, particularly with a compromised backbone. A guideline-directed and clinically rational decision is to rapidly escalate to a regimen that pairs DTG with a second high-barrier agent, usually a boosted protease inhibitor.^5^ In our scenario, escalation to dolutegravir twice daily with ATV/r regained viral suppression with modest immunologic recovery. Thirdly, specialist input is invaluable to construct durable salvage combinations, anticipate drug–drug interactions and tailor monitoring frequency. This approach reduces time spent in virological failure, the primary driver of resistance evolution.

The larger lesson is not to abandon DTG, which has transformed modern HIV management. Rather, we should optimise its utility. At an individual level, retaining DTG may preserve partial activity and reduce viral fitness, particularly when paired with a second high-barrier agent to regain suppression. At a programmatic level, DTG remains central because of its efficacy, tolerability and high genetic barrier. Strengthening adherence support, selecting patients for earlier genotyping and rapidly optimising regimens when failure is suspected will help preserve DTG durability. Case reports like ours complement large-scale programmatic data, providing nuanced, practical insights into managing emerging resistance.^1,2,3^ Dual-anchor therapy is a logical and systematic approach to suppressing viraemia when DTG resistance is confirmed or strongly suspected.^5^

## Teaching points

- Archived NRTI resistance can convert dolutegravir-based regimens into functional monotherapy, enabling selection of R263K.^1,4^
- Early resistance testing and rapid regimen escalation limit resistance accumulation and restore suppression.^2,5^
- Dual-anchor therapy with an integrase inhibitor plus a boosted protease inhibitor is a practical salvage option in complex cases.^5^

## Conclusion

Potent drugs such as DTG are susceptible to failure when combined with inactive companion drugs, and treatment escalation is delayed. Prompt viral suppression can be achieved when salvage therapy is utilised. Initiatives should promote earlier genotyping and access to specialist-guided management to preserve the durability of DTG for the foreseeable future.
